# A Novel Role of Silibinin as a Putative Epigenetic Modulator in Human Prostate Carcinoma

**DOI:** 10.3390/molecules22010062

**Published:** 2016-12-31

**Authors:** Ioannis Anestopoulos, Aristeidis P. Sfakianos, Rodrigo Franco, Katerina Chlichlia, Mihalis I. Panayiotidis, David J. Kroll, Aglaia Pappa

**Affiliations:** 1 Department of Molecular Biology and Genetics, Democritus University of Thrace, University Campus, Dragana, 68100 Alexandroupolis, Greece; i.anestopoulos@yahoo.com (I.A.); aristidis.sfakianos@postgrad.manchester.ac.uk (A.P.S.); achlichl@mbg.duth.gr (K.C.); 2 Redox Biology Center, School of Veterinary Medicine & Biomedical Sciences, University of Nebraska-Lincoln, Lincoln, NE 68583, USA; rodrigo.franco@unl.edu; 3 Department of Applied Sciences, Northumbria University, Newcastle Upon Tyne NE1 8ST, UK; m.panagiotidis@northumbria.ac.uk; 4 Department of Pharmaceutical Sciences, College of Science & Technology, Biomanufacturing Research Institute and Technology Enterprise (BRITE), North Carolina Central University, Durham, NC 27707, USA; davidjkroll@gmail.com

**Keywords:** silibinin, EZH2, PRC2, histone methylation, H3K27me3, DNMT, HDAC, epigenetics, prostate cancer

## Abstract

Silibinin, extracted from milk thistle (*Silybum marianum* L.), has exhibited considerable preclinical activity against prostate carcinoma. Its antitumor and chemopreventive activities have been associated with diverse effects on cell cycle, apoptosis, and receptor-dependent mitogenic signaling pathways. Here we hypothesized that silibinin’s pleiotropic effects may reflect its interference with epigenetic mechanisms in human prostate cancer cells. More specifically, we have demonstrated that silibinin reduces gene expression levels of the Polycomb Repressive Complex 2 (PRC2) members Enhancer of Zeste Homolog 2 (EZH2), Suppressor of Zeste Homolog 12 (SUZ12), and Embryonic Ectoderm Development (EED) in DU145 and PC3 human prostate cancer cells, as evidenced by Real Time Polymerase Chain Reaction (RT-PCR). Furthermore immunoblot and immunofluorescence analysis revealed that silibinin-mediated reduction of EZH2 levels was accompanied by an increase in trimethylation of histone H3 on lysine (Κ)-27 residue (H3K27me3) levels and that such response was, in part, dependent on decreased expression levels of phosphorylated Akt (ser473) (pAkt) and phosphorylated EZH2 (ser21) (pEZH2). Additionally silibinin exerted other epigenetic effects involving an increase in total DNA methyltransferase (DNMT) activity while it decreased histone deacetylases 1-2 (HDACs1-2) expression levels. We conclude that silibinin induces epigenetic alterations in human prostate cancer cells, suggesting that subsequent disruptions of central processes in chromatin conformation may account for some of its diverse anticancer effects.

## 1. Introduction

Prostate cancer (PCa) is the most frequently diagnosed malignancy in men and the sixth leading cause of cancer death worldwide [1,2]. The etiology of its incidence comprises various risk factors including aging, race, obesity, dietary habits, and environmental factors [3]. When localized, surgical prostatectomy and radiotherapy are effective treatments for most patients, while, for advanced and metastatic disease states, hormonal androgen deprivation therapy (ADT) has a high response rate [4,5]. However the majority of tumors evolve towards a hormone refractory state named castration-resistant prostate cancer (CRPC), which is associated with a high mortality [6,7]. Although the underlying molecular mechanisms have not been yet fully elucidated, genetic alterations, as well as epigenetic modifications, are implicated in the development and progression of the disease [8,9,10].

Silibinin is the principal active ingredient of silymarin (a mixture of seven flavonolignans and one flavonoid), extracted from seeds of milk thistle (*Silybum Marianum* L.), and is composed of two diastereoisomers (silybin A and silybin B) [11]. Silibinin has exhibited broad antineoplastic efficacy in a variety of cancer models including breast, lung, skin, oral, renal, and liver [12]. In PCa, its antitumor and chemopreventive activities have been associated with its interference with several determinants of tumor growth, such as cell cycle progression, apoptosis, invasion-metastasis, angiogenesis, transcription factors, and cell signaling [12,13]. This spectrum of such pleiotropic effects led us to investigate whether silibinin influences central processes of chromatin regulation that are implicated in PCa.

In recent years, the dominant role of Enhancer of Zeste Homolog 2 (EZH2), a chromatin-modifying enzyme, has been highlighted in the development and progression of PCa [14,15]. EZH2 is a member of the polycomb repressive group (PcG) of proteins, originally characterized in *Drosophila* as suppressors of homeotic genes required for segmentation [16]. In mammals, PcG proteins function in two distinct protein complexes; Polycomb Repressive Complex 1 (PRC1) and Polycomb Repressive Complex 2 (PRC2) [14]. PRC2 complex consists of EZH2, Embryonic Ectoderm Development (EED), and Suppressor of Zeste Homolog 12 (SUZ12). EZH2 is the catalytic subunit of the PRC2 complex and exerts methyltransferase activity *via* its Su(var)3-9, Enhancer-of-zeste, Trithorax (SET) domain, by trimethylating lysine (K)-27 residues of histone H3 (H3K27me3), resulting in chromatin condensation and gene repression [17,18,19]. EZH2 is also able to interact with DNA methyltransferases (DNMTs) [17] and recruit histone deacetylase proteins (HDAC) via the EED member [20], properties that are characterized as additional mechanisms of transcriptional repression [21,22]. EZH2 is overexpressed in hormone-refractory metastatic prostate cancer, associated with aggressiveness and elevated proliferation rates, while high expression levels in localized prostate cancers are correlated with elevated risk of recurrence after prostatectomy and poorer prognosis [23,24].

In the present study, we hypothesized that silibinin may interfere with the epigenetic cell machinery and focused on the function of the EZH2, often found deregulated in PCa. To this end, silibinin was shown to reduce, but not completely eliminate the expression of EZH2 in DU145 and PC3 human prostate carcinoma cell lines. Interestingly this reduction was associated with a dose-dependent loss of EZH2 phosphorylation (at serine-21), with an increase in (i) global histone H3 trimethylation at lysine-27 (Η3K27me3) and (ii) total DNMT activity and a decrease in (iii) HDAC1-2 expression levels. Finally we suggest that some of the observed anticancer effects of silibinin may be triggered via epigenetic mechanisms mediated by altered EZH2 function.

## 2. Results

### 2.1. Silibinin Suppresses PRC2 Complex Members’ Expression

First we evaluated the effect of silibinin on the mRNA levels of polycomb group PRC2 members, EZH2 and SUZ12, essential for the stability and activity of the PRC2 complex, as well as EED, required for the propagation of the H3K27me3 mark [25,26]. Androgen-independent DU145 and PC3 cells were incubated with increasing concentrations of silibinin (25–75 μg/mL), previously reported to be clinically achievable in Phase I Clinical trials [27].

**Figure 1:**
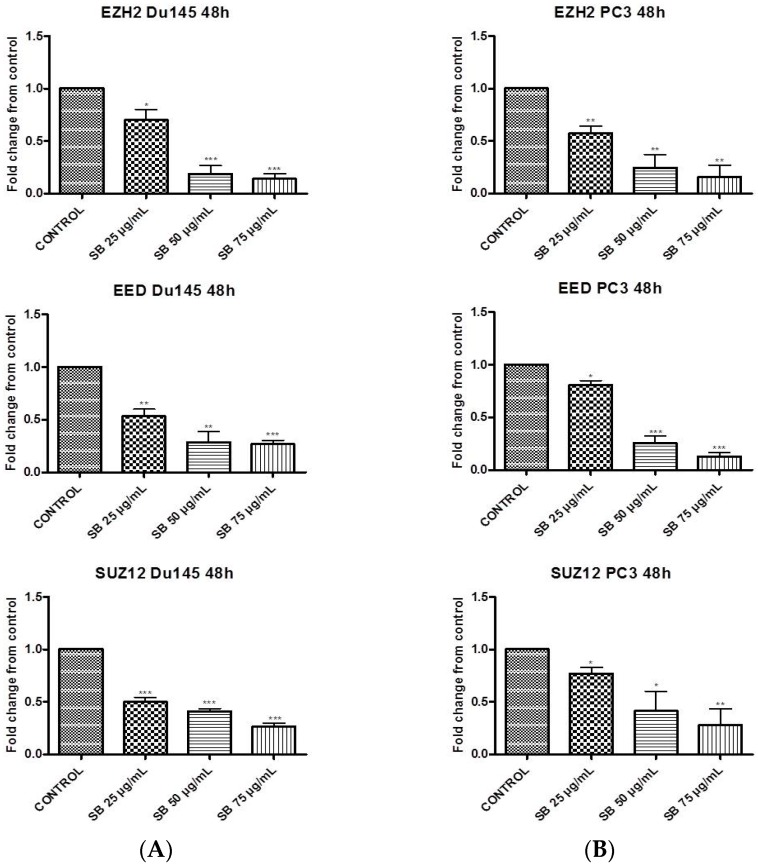
Silibinin suppresses the expression of Polycomb Repressive Complex 2 (PRC2) complex members. DU145 (**A**) and PC3 (**B**) cells were incubated for 48h with different concentrations of silibinin (25–75 μg/mL). After treatments, cells were harvested for Real Time Polymerase Chain Reaction (RT-PCR) analysis of Enhancer of Zeste Homolog 2 (*EZH2*), Suppressor of Zeste Homolog 12 (*SUZ12*) and Embryonic Ectoderm Development (*EED*) genes. The expression levels of target genes were normalized to β-actin (internal control). The results are presented as mean ± SD of three independent experiments. * *p* < 0.05; ** *p* < 0.01; *** *p* < 0.001.

According to the results, silibinin caused a significant dose-dependent reduction of all three members of the PRC2 complex, in both cell lines. More specifically, the reduction of EZH2, EED, and SUZ12 appeared to be greater in DU145 when compared to PC3 cells (Figure 1A,B respectively), as indicated by Real Time Polymerase Chain Reaction (RT-PCR) experiments.

### 2.2. Silibinin Reduces EZH2 Expression While It Increases Trimethylation Levels of Lys27 on H3 (H3K27me3)

**Figure 2:**
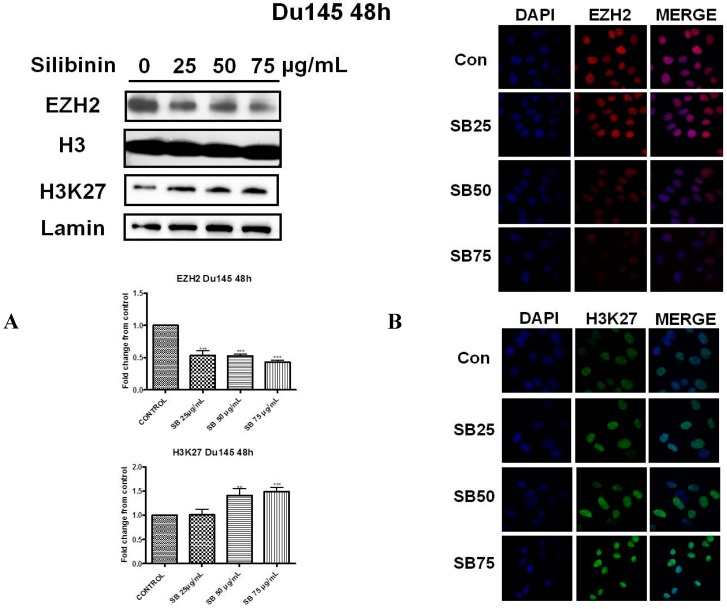
Silibinin reduces EZH2 expression while it increases trimethylation of Lys27 on H3 (H3K27me3). (**A**) DU145 cells were treated with different concentrations of silibinin (25–75 μg/mL) for 48 h. Nuclear extracts were analyzed for EZH2 and H3K27me3 by western blotting. Representative blots are shown. Equal protein loading was verified by stripping and re-probing the same membranes with histone H3 and lamin-β (2A upper panel). Quantification of EZH2 and H3K27me3 bands was performed by scanning densitometry (2A lower panel). The results are presented as the mean ± SD of three or more independent experiments. ** *p* < 0.01; *** *p* < 0.001; (**B**) DU145 cells were grown on coverslips in 6-well chamber plates for 24 h prior to treatments with various concentrations of silibinin (25–75 μg/mL) for 48 h. Cells were fixed and processed for staining with anti-EZH2 (2B upper panel) and anti-H3K27me3 (2B lower panel) antibodies, as described in ‘Materials and Methods’. Specific antibody staining was detected by fluorescein-conjugated secondary antibody and counterstained for DNA with 4′,6-diamidino-2-phenylindole (DAPI). The blue fluorescent staining column is for DAPI, the second red/green fluorescent column is for EZH2/H3K27me3 respectively, and the third column is for both images merged.

**Figure 3:**
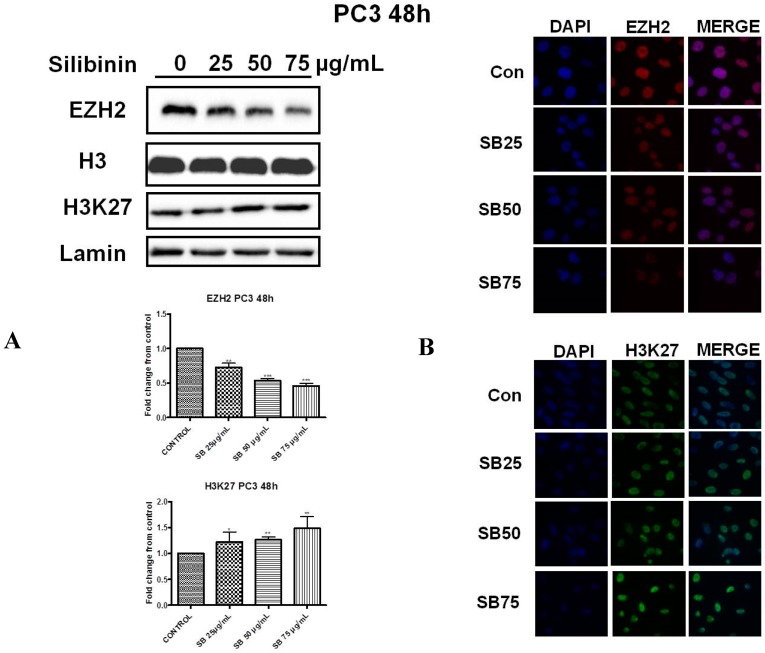
Silibinin reduces EZH2 expression while it increases trimethylation of Lys27 on H3 (H3K27me3). (**A**) PC3 cells were treated with different concentrations of silibinin (25–75 μg/mL) for 48 h. Nuclear extracts were analyzed for EZH2 and H3K27me3 by western blotting. Representative blots are shown. Equal protein loading was verified by stripping and re-probing the same membranes with histone H3 and lamin-β (3A upper panel). Quantification of EZH2 and H3K27me3 bands was performed by scanning densitometry (3A lower panel). The results are presented as the mean ± SD of three or more independent experiments. * *p* < 0.05; ** *p* < 0.01; *** *p* <0.001; (**B**) PC3 cells were grown on coverslips in 6-well chamber plates for 24h prior to treatments with different concentration of silibinin (25–75 μg/mL) for 48 h. Cells were fixed and processed for staining with anti-EZH2 (3B upper panel) and anti-H3K27me3 (3B lower panel) antibodies, as described in ‘Materials and Methods’. Specific antibody staining was detected by fluorescein-conjugated secondary antibody and counterstained for DNA with DAPI. The blue fluorescent staining column is for DAPI, the second red/green fluorescent column is for EZH2/H3K27me3, and the third column is for both images merged.

Next we investigated the effect of silibinin on EZH2 protein levels and those corresponding to trimethylation levels of H3 on Lys27 residues (H3K27me3), in both cell lines. It is well documented that one of the major activities of EZH2 is its ability to trimethylate histone H3 on Lys27 residues, which is associated with gene repression [17,18,19]. Western blot analysis in DU145 (Figure 2A, upper panel) and PC3 (Figure 3A, upper panel) cells, following 48h incubation with silibinin (25–75 μg/mL), revealed a significant decrease of EZH2 levels at each concentration tested. However this reduction was accompanied by a modest increase in global H3K27me3 methylation levels, in both cell lines, an observation that appears paradoxical given the observed reduction in EZH2 levels following treatment with silibinin.

To further confirm our results, we also employed immunofluorescence staining for detecting the cellular abundance of EZH2 and H3K27me3. As illustrated, silibinin suppressed the EZH2 specific immunofluorescence signal in DU145 (Figure 2B, upper panel) and PC3 (Figure 3B, upper panel) cells respectively. Upon examination of the intensity of the H3K27me3 immunofluorescence staining signals, increased tri-methylation levels were confirmed in both DU145 (Figure 2B, lower panel) and PC3 (Figure 3B, lower panel) cell lines after treatment with silibinin, which is in accordance with our western blot results.

### 2.3. Silibinin Decreases Phosphorylation of pAkt (ser473) with a Concomitant Suppression of pEZH2 (ser21) Expression Levels

**Figure 4:**
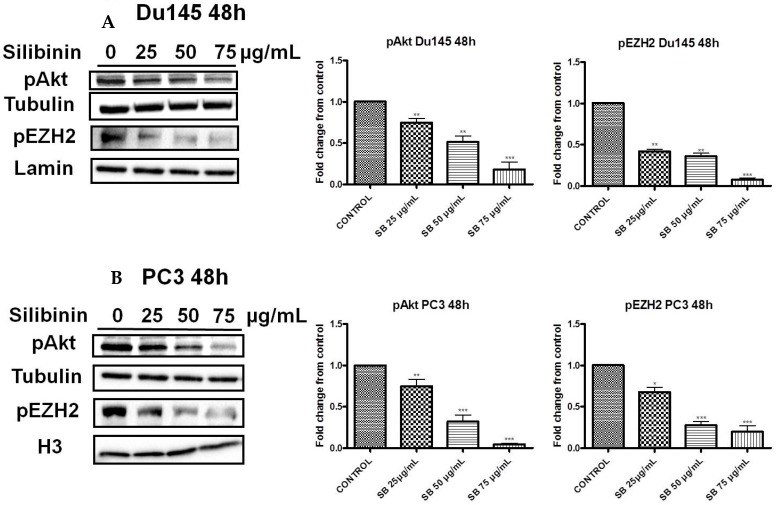
Silibinin decreases phosphorylation of pAkt (ser473) with a concomitant suppression of pEZH2 (ser21) levels. DU145 (**A**) and PC3 (**B**) cells were treated with different concentrations of silibinin (25–75 μg/mL) for 48 h. Cytosolic and nuclear extracts were analyzed for pAkt (ser473) and pEZH2 (ser21) by western blotting, respectively. Representative blots are shown. Equal protein loading was verified by stripping and re-probing the same membranes with tubulin and histone H3/lamin-β (left panel). Quantification of pAkt and pEZH2 bands was performed by scanning densitometry (right panel). The results are presented as the mean ± SD of three or more independent experiments. * *p* < 0.05; ** *p* < 0.01; *** *p* < 0.001.

Earlier studies have reported that EZH2 is a substrate of Akt. Specifically activated (phosphorylated) Akt can phosphorylate EZH2 (ser21 residues), thereby inhibiting its affinity for H3 and resulting in reduced H3K27 tri-methylation [28]. Τreatment of DU145 and PC3 cells with silibinin for 48 h led to a significant decrease of pAkt (ser473 residues) levels in a concentration dependent manner, which was accompanied by a strong reduction of pEZH2 levels in both PCa cell lines (Figure 4Α,B, left panels)*.* In this way, the silibinin-induced enhancement of H3K27 tri-methylation levels appears to be mediated by the suppression of Akt activity and the subsequent reduction of pEZH2 (ser21) levels in both cell lines.

### 2.4. Increased Trimethylation of Lysine 27 on Histone H3 by Silibinin Is Associated with Decreased pAkt Levels

**Figure 5:**
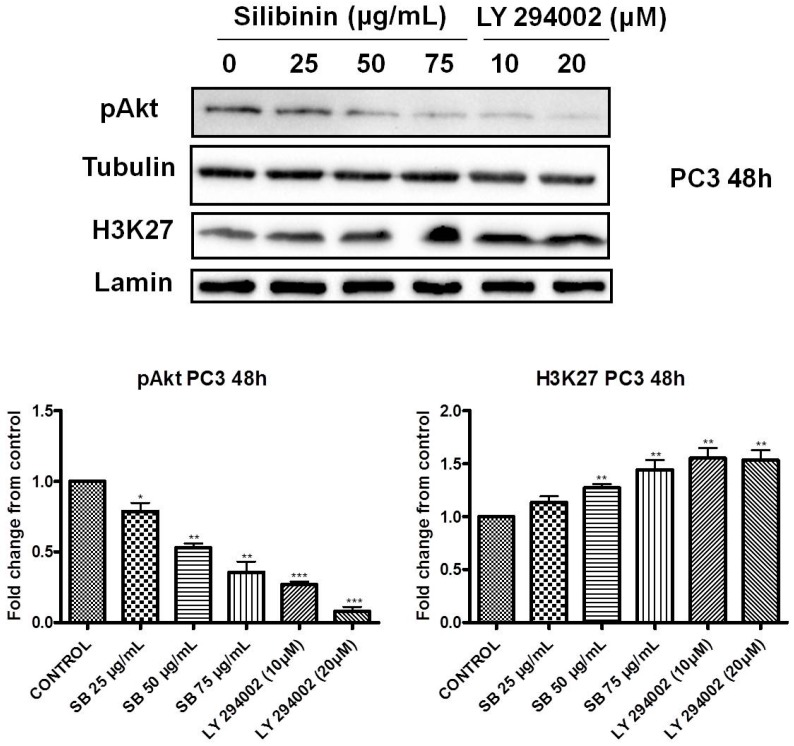
Increased trimethylation of lysine 27 on histone H3 by silibinin is associated with decreased pAkt levels. PC3 cells were treated with different concentrations of silibinin (25–75 μg/mL) and PI3K inhibitor LY294002 (10–20 μΜ) for 48 h. Cytosolic and nuclear extracts were analyzed for pAkt (ser473) and H3K27me3 by western blotting, respectively. Representative blots are shown. Equal protein loading was verified by stripping and re-probing the same membranes with tubulin and lamin-β (upper panel). Quantification of pAkt (ser473) and H3K27me3 bands was performed by scanning densitometry (lower panel). The results are presented as the mean ± SD of three or more independent experiments. * *p* < 0.05, ** *p* < 0.01, *** *p* < 0.001.

To check whether decreased levels of activated Akt are related to increased H3K27 tri-methylation levels, we treated PC3 cells with silibinin (25–75 μg/mL) or the PI3K inhibitor LY294002 (0, 10 and 20 μΜ) for 48 h and monitored the levels of pAkt and H3K27me3 by western blotting. We observed that both treatments with either silibinin or the inhibitor LY294002 resulted in decreased levels of pAkt and enhanced H3K27 trimethylation levels (Figure 5). Interestingly silibinin, at the highest concentration used, caused a reduction in pAkt with a concomitant increase in H3K27 trimethylation levels which was comparable with the effect caused by the concentration of 10 μM of the LY294002 inhibitor in PC3 cells.

### 2.5. Silibinin Causes a Modest Concentration-Dependent Increase in DNA Methyltransferase Activity

**Figure 6:**
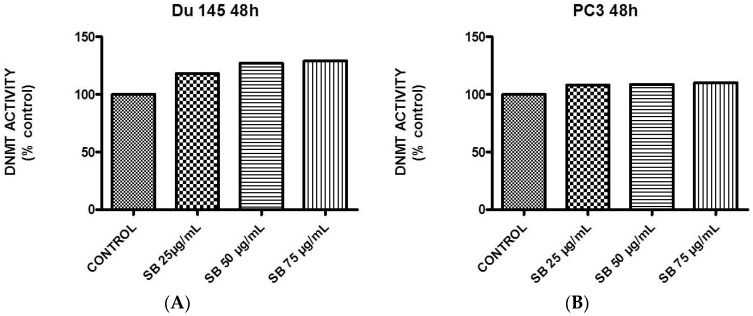
Silibinin induces a concentration-dependent increase in DNA methyltransferase activity. Logarithmically-growing DU145 (**A**) and PC3 (**B**) cells were treated with 25–75 μg/mL silibinin for 48 h and processed for quantification of global DNA methyltransferase activity.

EZH2 is thought to act as a transcriptional repressor, not only by tagging crucial histone residues, but also by recruiting DNA methyltransferases (DNMTs) that methylate CpG islands of promoter targets [17]. The effect of silibinin on overall DNMT activity was examined in DU145 and PC3 cells treated with increasing concentrations of silibinin (25–75 μg/mL) for 48 h. According to the results, a modest increase in DNMT activity was detected in both cell lines, with a more profound effect in the case of DU145 cells (Figure 6A,B).

### 2.6. Silibinin Causes Concentration-Dependent Decrease of HDAC1-2 Expression Levels

**Figure 7:**
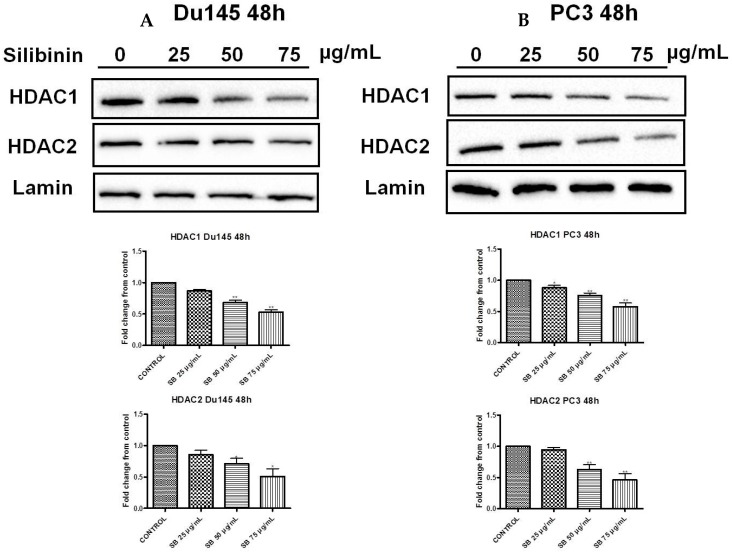
Silibinin induces a concentration-dependent decrease of HDAC1-2 expression levels. DU145 (**A**) and PC3 (**B**) cells were treated with different concentrations of silibinin (25–75 μg/mL) for 48 h. Nuclear extracts were analyzed for HDAC1 and HDAC2 by western blotting. Representative blots are shown. Equal protein loading was verified by stripping and re-probing the same membranes with lamin-β (upper panel). Quantification of HDACs1-2 bands was performed by scanning densitometry (lower panel). The results are presented as the mean ± SD of three or more independent experiments. * *p* < 0.05; ** *p* < 0.01.

Histone acetylation and deacetylation mediated by histone acetyltransferases (HATs) and histone deacetylases (HDACs) modifying enzymes are two additional histone post-translational modifications found deregulated in PCa [29,30]. In general, while acetylation enables transcriptional activity, HDACs remove acetyl groups leading to a condensed chromatin configuration [29]. Specifically, among their different classes, HDAC1-2 (Class I members) have been reported to be upregulated in PCa patients with high Gleason scores, while HDAC2 levels have been correlated with shorter relapse-free survival times following radical prostatectomy in PCa patients [31]. Our data indicate that treatment with silibinin significantly reduced HDAC1-2 levels in both DU145 (Figure 7A) and PC3 (Figure 7B) cell lines and thus confirm previous observations.

## 3. Discussion

PCa is one of the most complex and common noncutaneous cancers and is considered to be a leading cause of cancer deaths worldwide [29,32,33]. As in the majority of various cancer types, different genetic and epigenetic alterations are associated with the disease’s pathogenesis [8,34]. Gene regulation in PCa is governed at the epigenetic level by three distinct (but not isolated) mechanisms: (i) DNA methylation (hyper/hypo); (ii) histone modifications, and (iii) microRNA deregulation [3,35].

EZH2, a methyltransferase enzyme and part of the PRC2 complex, represses gene expression via methylation of lysine 27 histone H3 residues (H3K27me3). EZH2 is highly expressed not only in PCa [23], but also in a variety of cancerous tissues such as breast, bladder, gastric, lung, liver, and ovary [24,33,36,37,38]. Because of their reversibility, epigenetic marks are attractive targets for cancer therapy. In fact, in recent years, there has been a considerable effort in this context by utilizing various phytochemicals (especially flavonoids) as therapeutic approaches for the prevention and treatment of various types of cancer [39]. To this end, silibinin has been shown to exert significant anti-neoplastic activity in a variety of tumor models including breast, lung, colon, and bladder [40]. In PCa, silibinin exerts multiple effects on cell growth arrest and apoptosis [12,13], thus allowing it to enter clinical trials for the treatment of the disease [27,41].

In the present study, we attempted to identify whether the previously reported pleiotropic effects of silibinin in PCa are mediated, at least in part, via its interference with major epigenetic marks, known to be implicated in the progression of the disease. Our initial results revealed that silibinin was able to reduce not only EZH2, but also EED and SUZ12 expression levels (Figure 1A,B). These are members of the PRC2 complex, which are essential for the stability-integrity of the complex and the propagation of the H3K27me3 mark [26]. However, although levels of EZH2 expression in DU145 and PC3 cells were reduced following treatment with silibinin, an increase in global levels of H3K27me3 (Figure 2 and Figure 3) was observed. The inverse relationship between EZH2 and H3K27me3 levels is thought to be related to pAkt levels, given that EZH2 is a substrate of Akt and activated (phosphorylated) Akt is responsible for the phosphorylation of EZH2 (ser21 residues) [28]. This, in turn, reduces the affinity of EZH2 for histone H3, thus resulting in decreased levels of H3K27me3 [28]. In this study, silibinin was able to reduce not only pAkt but also to diminish pEZH2 levels (quite complete loss) in a concentration dependent manner (Figure 4A,B). So while silibinin suppresses EZH2 abundance, its potent effect on reducing EZH2 phosphorylation appears to be associated with an increase in H3K27me3 marks in DU145 and PC3 cells. Moreover the observed reduction of pAkt levels by silibinin followed the exact same pattern with that observed with LY294002 (a specific PI3K/Akt inhibitor) (Figure 5).

A number of studies have demonstrated that, in prostate carcinoma cells, silibinin causes hypophosphorylation of retinoblastoma (Rb) protein, an increase in Rb-E2 factor (E2F) complexes, and substantial decreases in some of the E2F family members [42,43]. Silibinin also induces increased expression of the insulin-like growth factor binding protein-3, (IGFBP-3) causing blunting of the insulin growth factor-1 (IGF-1) signaling pathway, as evidenced by reduced phosphorylation of the insulin receptor substrate-1, (IRS-1) in PC3 cells [44]. It is interesting to note that E2F is a positive regulator of EZH2 [45] and IGFBP-3 has emerged as a PRC2-regulated gene [46]. In a study using the EZH2 inhibitor 3-deazaneplanocin A (DZNep), it was found that its treatment depleted breast carcinoma cells of the PRC2 complex members (EZH2, SUZ12, and EED),and subsequently reactivated expression of IGFBP-3 [46]. Resveratrol, a natural polyphenolic flavonoid, was recently found to suppress myofibroblast activity of human buccal mucosal fibroblasts (fBMFs), responsible for the pathogenesis of the precancerous condition oral submucous fibrosis (OSF), through down-regulation of the zing finger E-box binding Homeobox 1 (ZEB1). The inhibition of ZEB1 in fBMFs by resveratrol was mediated by epigenetic mechanisms dictated by the upregulated expression of miR-200c and the enhancer of zeste homolog 2 (EZH2), as well as H3K27me3. Resveratrol increased the binding of H3K27me3 to the ZEB1 promoter, causing its down-regulation and the suppressed myofibroblast activity of fBMFs [47]. A ZEB1-miR-375-YAP1 pathway was recently found to regulate epithelial plasticity in prostate cancer [48], while ZEB1 together with STAT3 were identified as key molecules altered at the early stages of prostate carcinogenesis [49]. Remarkably silibinin was able to reverse epithelial-to-mesenchymal transition (EMT) in metastatic prostate cancer cells by down-regulating the two major EMT regulators, ZEB1 and SLUG transcription factors [50], while its role in inhibiting STAT3 signaling is well documented [51]. Therefore it will be interesting to elucidate if silibinin-mediated alterations in gene expression in prostate carcinoma cells are due to decreased EZH2 abundance or altered EZH2 function.

The way by which perturbations in H3K27me3 levels are associated with cancer prognosis, diagnosis and therapeutic outcome remains puzzling. For instance, global loss of H3K27me3 levels in clinical samples has been associated with poor prognosis in renal, pancreatic, breast, ovarian, and non-small cell lung cancer (NSCLC) [52,53,54], while overexpression was related to malignancy and worse prognosis in esophageal, nasopharyngeal, and liver cancer patients [55,56,57], thus indicating that the function of H3K27me3 is cancer-type specific [58]. In PCa, there are different reports concerning H3K27me3 levels and prognosis. For example, in a clinical study, H3K27me3 global levels were higher in metastatic (mPCa) and CRPC samples, compared to localized and normal prostate samples. According to the authors, the small size of the study together with the inability of obtaining follow-up information for mPCA and CRPC patients were the two main reasons as to why there could not be observed a correlation between H3K27me3 levels and different clinicopathological characteristics [59]. Post-therapy, global plasma H3K27me3 levels were found to be reduced in metastatic compared to localized or local advanced PCa patients, but there were no data for H3K27me3 levels before treatment [54,60]. In two additional studies conducted by the same research group, H3K27me3 levels were found to be significantly enriched at promoter sites of different genes, like retinoid acid receptor β2 (RARbeta2), estrogen receptor a (ERa), progesterone receptor (PGR), and repulsive guidance molecule A (RGMA) in prostate peritumoral (PPT) and/or PCa tissues [61,62]. In addition, other studies have shown that global levels of H3K27me3 were decreased in prostatic intraepithelial neoplasia (PIN) as well as in metastatic and high-grade tumors. Furthermore areas of overexpression of EZH2 staining did not correlate with increased H3K27me3 marks, as would be expected. According to the authors, low H3K27me3 levels are regulated, at least in part, to myelocytomatosis oncogene (MYC)overexpression in PCa (known to be a positive regulator of EZH2 expression), although data on histone lysine methyltransferase activity or pEZH2 levels that effect EZH2 affinity for H3 histone were not measured [63,64]. Nevertheless it remains unclear how increased levels of MYC-EZH2 are accompanied by low H3K27me3 levels in PCa, but it is reasonable to assume discrepancies by (i) the different experimental procedure followed; (ii) the nature of measurement (i.e., global, vs. promoter-specific levels, etc.); and (iii) the biological material used (cell lines, samples, body fluids, etc.). Moreover the lack of a reported direct correlation between H3K27me3 and total/phosphorylated EZH2 levels should not be ruled out in justifying the controversial results mentioned above.

The effects of silibinin on global DNMT activity revealed a modest increase in both cell lines (Figure 6), suggesting the ability of EZH2 to recruit DNMTs that methylate CpGs in promoter regions as an additional mechanism of gene repression [17]. However how increased global methylation contributes towards its observed anti-neoplastic activity remains to be further elucidated. For instance, it will be worth evaluating, in future experiments, the effect of silibinin on specific DNMT enzymes (e.g., DNMT1), which appear to be upregulated in PCa. To this end, it will be of paramount importance to assess the net effect of commercial DMNT inhibitors as mono versus combined (adjuvant) therapy with silibinin. Interestingly a recent report showed that, although 5-azacytidine (5-AZA) reduced DNMT1 expression levels in PC3 and DU145 cells, this result was associated with EMT and cancer stem cell (CSC) phenotypes, events likely to be counterproductive when treating PCa [65].

Furthermore, in the present study, we have determined that silibinin inhibited HDACs 1-2 levels in a concentration-dependent manner (Figure 7), thus highlighting its ability to regulate gene expression. HDACs are implicated in the regulation of gene expression by removing acetyl groups from histone residues. In that way, DNA is tightly wrapped by histones, and, as a result, specific genes expression is inhibited. HDACs1-2 (Class I members) are found overexpressed in PCa [31]. Various studies have implicated the ability of silibinin to interfere with specific epigenetic marks including (i) the inhibition of DNMT activity in SW480 and SW620 colon adenocarcinoma cells, without affecting HDAC levels [66]; (ii) the induction of acetylated H3 and H4 histones in Huh7 hepatoma cells and a hepatocellular carcinoma xenograft model [67,68], as well as in non-small lung cancer cells (NSCLC) H1299 and xenograft models [69]; and finally (iii) the reduction of HDACs 1-2-3 levels when treated either alone or in combination with HDAC inhibitors such as trichostatin (TSA) and suberoylanilide hydroxamic acid (SAHA, Vorinostat) [45,69]. To this end, NSCLC cell treatment with low doses of silibinin (i.e., 3.75–12.5 μM) in combination with TSA and 5-AZA, restored the expression of E-cadherin and downregulated the expression of ZEB1 (its transcriptional repressor) [70]. Interestingly increased H3K27me3 has been associated with the down-regulation of ZEB1 expression [71]. Overall it remains to be further elucidated if the expression of other HDAC members is modulated by silibinin treatment alone or in the context of an adjuvant scheme.

## 4. Materials and Methods

### 4.1. Cell lines and Treatments

Human prostate cancer cell lines DU145 and PC3 were purchased from the American Type Culture Collection (ATCC, Manassas, VA, USA). DU145 and PC3 cells were cultured in a DMEM-F12 medium supplemented with 10% fetal bovine serum (FBS), 100 U/mL penicillin, and 100 μg/mL streptomycin. Cells were incubated in a humidified 5% CO_2_/37 °C incubator. All the experiments were performed on logarithmically growing cells. Cells were seeded at a density of 1 × 10^6^ cells per dish (100 mm) for western blot, RT-PCR, and DNMT activity analysis, while for immunofluorescence experiments 2 × 10^5^ cells were seeded in 6-well culture dishes. At 70%–80% confluency, cells were treated with increasing concentrations of silibinin (25–75 μg/mL) for 48 h. Following treatments, the culture medium was aspirated and the cells were collected with a gum rubber-scrapping device.

### 4.2. Reagents and Antibodies

The DMEM-F12 culture mediums, along with other cell culture materials (FBS, antibiotics, trypsin) were from Biochrom (Berlin, Germany), Biosera (East Sussex, UK), Gibco (Life Technologies, Carlsbad, CA, USA), and Sigma-Aldrich Co. (Taufkirchen, Germany). Silibinin was obtained from Sigma-Aldrich Co. (Taufkirchen, Germany). Polyvinylidene difluoride (PVDF) membranes were purchased from Millipore (Bedford, MA, USA). Chemiluminescence reagents were from Thermo Scientific (Rockford, IL, USA). For protein estimation, the Pierce™ BCA Protein assay from Thermo Scientific (Rockford, IL, USA) was used. The primary antibodies used in the experiments included; anti-EZH2 (D2C9), anti tri-methyl Histone H3 (Lys 27), anti-phospho-Akt (ser473), anti-Lamin B2 (P8P3U), anti-HDAC1 (10E2), anti HDAC2 (3F3) obtained from Cell Signaling Technology (Boston, MA, USA), anti-phosphoEZH2 (ser21) obtained from Bethyl Laboratories (Inc., Montgomery, TX, USA); anti-histone H3 and anti-β tubulin were purchased from Sigma-Aldrich Co. (Taufkirchen, Germany). Goat anti-rabbit and anti-mouse IgG horseradish peroxidase conjugated antibodies were from Millipore (Bedford, MA, USA), while CF405S Goat anti-rabbit and CF488A Goat anti-rabbit antibodies for immunofluorescence were obtained from Biotium (Hayward, CA, USA). DAPI was from Sigma-Aldrich Co. (Taufkirchen, Germany) and Mowiol mounting medium for immunofluorescence was from Calbiochem EMD Biosciences (San Diego, CA, USA). Proteinase inhibitors were from Sigma-Aldrich Co. (Taufkirchen, Germany) and phosphatase inhibitors were from Cell Signaling Technology (Boston, MA, USA). Primers, dNTPs, and Platinum SYBR green were from Invitrogen (Life Technologies, Carlsbad, CA, USA), while random hexamers and Prime Script Reverse Transcriptase were from Takara (Shiga, Japan). For an estimation of total DNMT (DNA Methyl Transferase) activity experiments, the DNMT Activity Quantification Kit (Fluorometric) was purchased from Abcam (Cambridge, UK).

### 4.3. Protein Extraction, Cell Lysates Preparation and Western Immunoblotting

At the end of the treatments, PBS-washed cells were lysed with a lysis buffer (10 mM HEPES, 1 0 mM KCl, 0.1 mM EDTA, 1.5 mM MgCl_2_, 0.2% NP40), supplemented with proteinase inhibitors (0.5 mM phenylmethylsulfonylfluoride, 2.5 μg/mL aprotinin, 2.5 μg/mL pepstatin A, 2.5 μg/mL leupeptin), and a phosphatase inhibitor cocktail. Lysates were incubated for 30 min at 4 °C, vortexed every 10 min and centrifuged at 1000× *g* for 10 min, to prepare cytosolic (supernatant) and nuclear (pellet) fractions. Protein quantification was performed with Pierce™ BCA Protein Assay kit, according to the manufacturer’s instructions. Cytosolic (40 μg) and nuclear (15 μg) fractions-samples were prepared and subjected to SDS-PAGE on 8%, 10% and 12% Tris-Glycine gels. The resultant separated proteins were transferred to PVDF membranes, while non-specific sites were blocked with 5% non fatdry milk in 150 mM NaCl, 100 mM Tris pH 7.5, and 0.1% (*v*/*v*) Tween-20, at room temperature for 2 h. The membranes were then hybridized overnight at 4 °C with the primary antibodies at different dilutions against anti-EZH2 (1/1000), anti-trimethyl histone H3 (Lys-27) (1/1000), anti-pAkt (Ser-473) (1/1000), anti-H3 (1/5000), anti-β tubulin (1/20,000), anti-Lamin β (1/1000), anti-pEZH2 (Ser-21) (1/500), anti-HDAC1 (1/1000) or anti-HDAC2 (1/1000). Then membranes were incubated with secondary horseradish peroxidase conjugated anti-rabbit and anti-mouse IgG antibodies (1/5000) for 1 h at room temperature, while immunoblot bands were developed using Chemidoc MD Imaging System (Biorad, Hercules, CA, USA). When necessary, the blots were stripped with a stripping buffer (62.5 mM Tris-HCl pH 6.7, 2% SDS, 6 μL/mL 2-mercaptoethanol) and reprobed with an alternate antibody. To ensure equal protein loading, each membrane was stripped and re-probed with anti β-tubulin, anti-H3, and anti-lamin B2. Band intensity was quantified by scanning densitometry using Image J software (1.44n, National Institute of Health, Bethesda, MD, USA).

### 4.4. RT-PCR Methodology

Total RNA was extracted from cultured cells using Trizol Reagent. For cDNA synthesis, a mixture (10 μL) of 4.5 μg of RNA, 1 mM dNTPs, and 50 pmol of random hexamers was incubated at 65 °C for 5 min. Next 240 units of Prime Script Reverse Transcriptase, 4.0 μL of the reverse transcriptase buffer (5×), and 0.25 μL of human placental ribonuclease inhibitor (40 U/μL) were added to a final volume of 20 μL and incubated at 30 °C for 10 min, 42 °C for 50 min, and a further 15 min at 70 °C. RT-PCR was performed by using a Step One instrument (Applied Biosystems, Waltham, MA, USA), utilizing the Platinum SYBR Green Mastermix according to the manufacturer’s instructions. Overall the following parameters were used: Step 1, 2 min at 50 °C; Step 2, 2 min at 90 °C; Step 3, 15 s at 95 °C; and Step 4, 30 s at 60 °C. Step 3 and 4 were repeated 40 times, while melt curve analysis was performed by heating for 15 s at 95 °C and cooling for 1 h at 60 °C, followed by heating at 95 °C for 15 min. Primers were designed with the Primer Express 3.0 Software (Applied Biosystems) and were as follows: *EZH2:* For; 5′ AATGGAAACAGCGAAGGATACAG, Rev; 5′ GCGCAATGAGCTCACAGAAG: *SUZ12:* For; 5′ TTTAGTCGCAACGGACCAGTT, Rev; 5′ CGTTTTGGCCTGCACACA: *EED:* For; 5′ GAACGCCCTGATACACCTACAAA, Rev; 5′ CCCTTTCCCCAACTTTTCCA: *β-actin:* For; 5′ GCGCGGCTACAGCTTCA, Rev; 5′ CTTAATGTCACGCACGATTTCC. Finally gene expression was normalized to β-actin using the 2^−ΔΔ*C*^T method.

### 4.5. Confocal Immunofluorescence Protocol

DU145 and PC3 cells were grown on glass coverslips in 6-well culture dishes. At the end of treatments, cells were fixed with 4% paraformaldehyde (PFA)/PBS pH 7.4 for 10 min. PFA was neutralized by the addition of 1 M glycine/pH 8.5 and cells were permeabilized by adding PBS pH 7.4/1% Triton-X for 5 min. Blocking was conducted by 5% (*w*/*v*) BSA/PBS pH 7.4 for 15 min and cells were incubated for 1.5 h at room temperature with anti-EZH2 (1/200) and anti-trimethyl histone H3 (Lys-27) (1/800). Cells were washed with PBS and incubated with appropriate secondary antibodies CF405S and CF488A (1/1000) for 1 h at room temperature. Subsequently DNA was counterstained with DAPI (1 μg/mL) for 5 min, washed with PBS and coverslips were mounted with Mowiol mounting medium (Calbiochem EMD Biosciences, San Diego, CA, USA) prior to observation. Cell images were obtained with a 60× lens and an Andor Ixon 885 digital camera on a customized Andor Revolution spinning disc confocal system unit around stand (IX81 Olympus, Belfast, UK) (Cibit Facility MBG-DUTH). For image acquisition, Andor IQ 2.7.1 software (Belfast, UK) was used, while optical sections were recorded every 0.3 μm. For images analysis, Image J software was used.

### 4.6. DNA Methyltransferase Activity Assay

Logarithmically-growing PC3 and DU145 cells were incubated with 25–75 μg/mL of silibinin for 48 h. At the end of the incubation period, nuclear extracts were prepared, and the global DNA methylatransferase activity content was measured using the DNMT Activity Quantification Kit (Fluorometric) (Abcam), according to the manufacturer’s instructions.

### 4.7. Statistical Analysis

Data are represented as the mean ± SD of three or more independent experiments. Statistical differences were evaluated by Student’s *t-*test. All statistical analyses were performed using Graph Pad Prism (GraphPad software, San Diego, CA, USA) while a value of *p* < 0.05 was considered statistically significant.

## 5. Conclusions

In the present study, silibinin was shown to reduce expression levels of the PRC2 complex members. More specifically, silibinin significantly inhibits the expression of the major regulatory protein EZH2, which is otherwise overexpressed in PCa. Such inhibition occurred at both total and phosphorylated states and was accompanied by the enhancement of global H3K27me3 levels, concomitant with a reduction of HDACs 1-2 levels. We conclude that silibinin interferes with key epigenetic pathways in regulating gene expression and thus offer a novel justification of how it exerts such pleiotropic antitumor activities against various types of cancer including PCa. Further work is required to dissect the interplay between silibinin and its potential target(s) in order to identify the downstream signaling cascades responsible for transducing its multipotent efficacies in various types of human cancer.
